# ENT foreign bodies: profile of the cases seen at a tertiary hospital emergency care unit

**DOI:** 10.5935/1808-8694.20130128

**Published:** 2015-10-08

**Authors:** João Mangussi-Gomes, José Santos Cruz de Andrade, Rafaella Caruso Matos, Eduardo Macoto Kosugi, Norma de Oliveira Penido

**Affiliations:** a M.D. (ENT and HNS Resident Physician, Federal University of São Paulo, Paulista School of Medicine (UNIFESP-EPM)); b Medical Student (Medical Student, Federal University of São Paulo, Paulista School of Medicine (UNIFESP-EPM)); c Ph.D. (Head Preceptor, ENT and HNS Residency, Federal University of São Paulo, Paulista School of Medicine (UNIFESP-EPM)); d Post-doctoral degree holder (Adjunct Professor, Department of Otorhinolaryngology and Head and Neck Surgery, Federal University of São Paulo, Paulista School of Medicine (UNIFESP-EPM))

**Keywords:** epidemiology, foreign bodies, myiasis, otorhinolaryngology

## Abstract

Individuals often seek help with foreign bodies (FB) in their ears, noses, and throats. Proper recognition, study, and management of foreign bodies is required to prevent complications.

**Objective:**

To analyze the profile of the patients seen for FB at a reference otorhinolaryngology emergency care unit between February of 2010 and January of 2011.

**Method:**

Cross-sectional retrospective historical cohort study based on digitized patient charts.

**Results:**

FB accounted for 827 cases and 5.3% of all patients seen in the ENT emergency unit. Children were affected more frequently, particularly when aged 8 and under. No statistically significant differences were seen between genders. Foreign bodies were mostly located in the ears (64.4%), followed by the nasal fossae (19.5%), and the oropharynx (8.9%). Complications were seen in 4.5% of the cases, and 4.4% required general anesthesia to have the FB removed.

**Conclusion:**

In our ENT practice, foreign bodies were more commonly seen in children; the ears were the preferential site of occurrence. Complication rates and use of general anesthesia were low in our practice. It should be stressed that ENT foreign bodies need to be properly managed so as to avoid complications.

## INTRODUCTION

Foreign bodies (FB) in the ears, nose or throat are a common occurrence in otorhinolaryngology (ENT) emergency services. Foreign bodies have been estimated to account for approximately 11% of the cases seen in ENT services1., 2., 3.. Severe complication may occur in as many as 22% of the cases - which speaks to the morbidity associated with foreign bodies; therefore, foreign bodies should be properly recognized, studied, and managed^4^.

Although FB removal is usually a simple procedure, its potential complications call for the aid of an ENT physician. Successful removal relies on a number of factors, including the location of the FB, what it is made of, the physician's dexterity, the equipment available, and patient cooperation5., 6.. FB removal is often carried out in an operating room, with the patient under sedation or general anesthesia4., 7.. Delayed treatment has been correlated with larger and more severe lesions, in addition to more complications^7^.

This study aimed to review the epidemiological characteristics of patients seen for FB in the ENT emergency service of a tertiary university hospital between February of 2010 and January of 2011.

## METHOD

This cross-sectional retrospective historical cohort study was based on the data of the patients seen in the ENT emergency service of a tertiary university hospital between February of 2010 and January of 2011.

The digital charts of patients seen for foreign bodies were used in data collection. The following data points were captured: date the patient was seen, age, gender, type and time for which the FB had been lodged, chosen clinical approach, complementary tests, complications and use of antibiotics, referral to other specialized services, and removal in an operating room.

All the cases of FB seen at the ENT emergency unit were included. Patient charts in formats other than digital and charts with incomplete patient information were excluded.

Patients were grouped based on the location of the FB: ears, nasal fossae, oropharynx, and larynx. Foreign bodies in the esophagus are not seen in the ENT service, but are relevant to the study as they are considered in the differential diagnosis. Therefore, they were also include in the study. Cases of myiasis were analyzed separately because of their peculiarities. This study was approved by the Ethics Committee of our institution and given permit 0081/10.

## RESULTS

The ENT emergency unit saw 15,640 cases within the period of time considered in the study. Foreign bodies accounted for 827 visits, or 5.3% of all cases.

**Graph 1:**
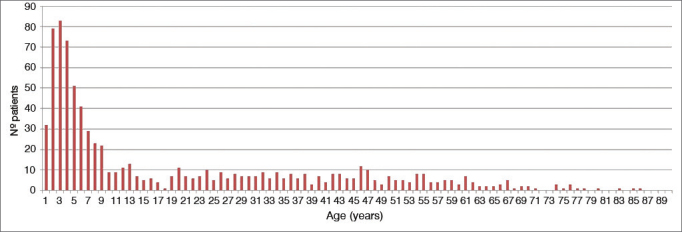
Description of patients seen for foreign bodies - age distribution (years).

**Graph 2:**
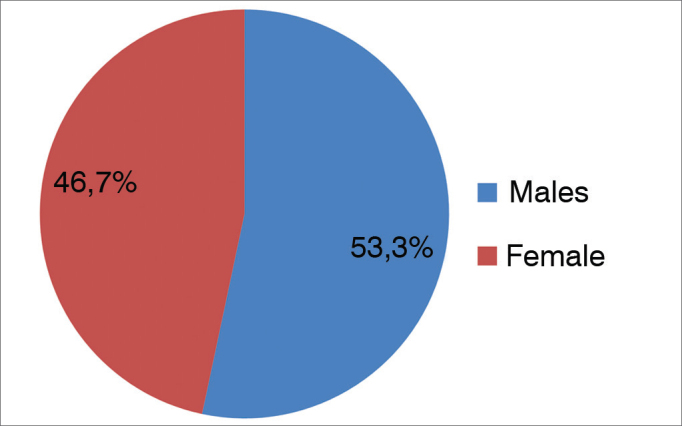
Description of patients seen for foreign bodies - gender.

Patients had a mean age of 19.8 years and a median age of 8 years. Incidence was greater among individuals aged 8 and under; peak incidence was seen at 3 years of age. However, foreign bodies were observed in patients of all ages (Graph 1). Of the 827 patients included in the study, 386 were females (46.7%) and 441 were males (53.3%), yielding a male-to-female ratio of 1.14:1.00 (Graph 2). Most foreign bodies (94.8%) were located in the ear, nose or throat.

**Graph 3:**
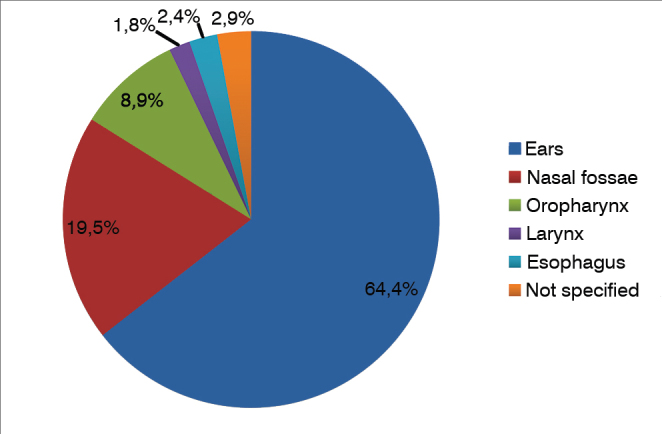
Location of foreign bodies.

**Graph 4:**
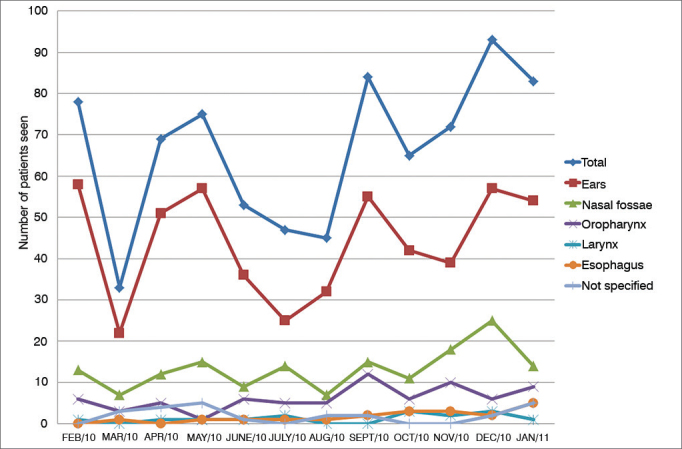
Distribution of cases of foreign body throughout the period analyzed in the study by location.

Graph 3 describes the location of foreign bodies in the group of patients included in the study. They were mostly located in the ears (64.4%), followed by the nasal fossae (19.5%), and the oropharynx (8.9%). FB location was not specified in 2.9% of the cases. The temporal distribution of cases of FB is shown in Graph 4.

Only 49 (5.9%) underwent complementary testing. The most frequently performed tests were X-ray imaging and upper digestive endoscopy, in 69.4% and 20.4% of the cases, respectively.

**Graph 5:**
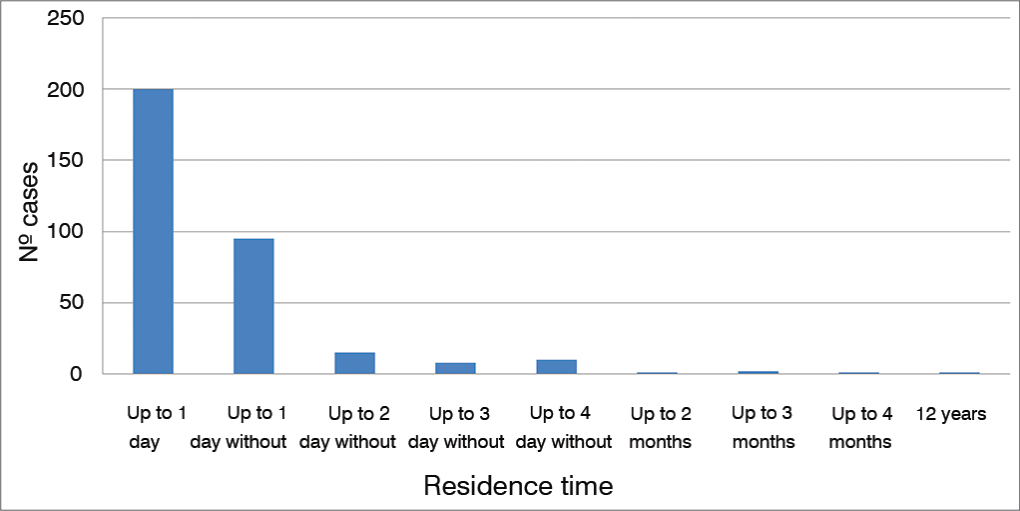
Estimated mean time of residence of foreign bodies.

The type of FB varied depending on the site of occurrence. Cotton fragments were the most common type of FB found in the ears. However, insects and beans were also frequently seen. Inanimate objects were the norm in the nasal fossae, with beans ranking atop all FB types. In the oropharynx and larynx, fish and chicken bones topped the list. Table 1 shows the most common types of FB for each site of occurrence. Graph 5 describes the time for which patients had foreign bodies in their ENT cavities.

**Table 1:** Most commonly seen FB types and their locations.

Location	FB Type	Frequency
	Cotton fragments	24.06%
Ear	Insects	22.56%
	Beans	8.65%
	Beans	17.07%
Nasal fossae	Sponge fragments	9.76%
	Plastic parts	7.32%
Oropharynx	Fish bones	70.69%
	Chicken bones	15.52%
Larynx	Chicken bones	45.45%
	Fish bones	18.18%

Myiasis was seen in six patients, or 0.7% of the cases seen in the ENT emergency unit. Although only six patients were accounted for with myiasis, 21 visits to the ENT emergency unit were recorded within the period of the study. Five of the six cases of myiasis were seen in males. All were adults, aged between 20 and 42 years. Ear myiasis was seen in five of the six cases. One patient had nasal myiasis.

Complications were more frequent in patients with foreign bodies in their ears. Twenty-three patients (2.8% of all cases) had the following ear complications: acute external otitis (12), laceration/bruising of the outer ear canal (5), tympanic membrane perforation (4), and acute otitis media (2). Only 14 patients (1.7% of all cases) with nasal foreign bodies had complications; 13 had acute rhinosinusitis and one had a perforated nasal septum. Therefore, complications were seen in 4.5% of the studied patients. Thirty-six patients (4.4%) required general anesthesia or sedation to have the FB removed.

## DISCUSSION

This study considered the patients seen for FB and myiasis in the ENT emergency unit of a tertiary university hospital for 12 consecutive months. The 827 cases of FB accounted for 5.3% of all patients seen in the ENT emergency unit. According to the literature, foreign bodies could account for as many as 11%1., 2., 3. of the cases. Yet, the absolute number of patients seen for FB in one year was considerably greater than the figures reported by other authors, thus reflecting the number of cases included in our analysis1., 2., 7..

Foreign bodies were more prevalent in children, and 50.1% of the patients were aged 8 or under. Male individuals were the majority by a slight difference (53.3%). These findings are in agreement with the literature and reports of FB being more common in children around six years of age1., 4., 7.. Cases in adults have also been reported. According to some authors, foreign bodies are commonly seen in adults, particularly in individuals with special needs8., 9..

ENT foreign bodies were the most common complaint reported by the patients seen in our center (94.8%). Other authors reported similar rates of occurrence of ENT foreign bodies6., 10.. This is explained, in most of the cases, by how easy it is to identify such foreign bodies and for the patient to report the issue to his/her caregiver^1^.

Foreign bodies were mostly located in the ears (64.4%), followed by the nose (19.5%), and the oropharynx (8.9%), as similarly reported in the literature3., 10.. Some authors suggested the following specific order of frequency and location of foreign bodies: ears, nose, pharynx, esophagus, and tracheal bronchial tree^10^.

Complementary tests are rarely needed in FB patients. Direct visualization during physical examination is usually enough to identify and locate foreign bodies. In this study, 49 patients (5.9%) had to undergo complementary tests, and 69.4% of them had simple X-ray images taken. X-ray imaging may help identify radio-opaque foreign bodies, but it is not useful in the diagnosis of radiolucent objects such as fish and chicken bones. According to some authors, complementary tests should only be performed in patients suspected with foreign bodies when careful physical examination and nasal and laryngeal endoscopy failed to produce additional evidence1., 5..

Foreign bodies have their social and geographic peculiarities. For example, cases involving cotton seeds and fragments are more commonly seen in developing countries. These were also the most commonly found objects in the ears and noses of the patients included in this study. Conversely, small plastic parts are the most frequent finding in developed countries9., 11..

Quick atraumatic removal of foreign bodies is a real challenge for ENT physicians. Therapeutic success depends on a number of factors, but there is no strong evidence to indicate one specific removal method over others^5^. It is known, however, that the permanence of foreign bodies in ENT cavities for over 72 hours and repeated attempts to remove the FB increase the risk of complications, both iatrogenic and not, in addition to considerably reducing the chances of success1., 2., 12..

In our study, most foreign bodies were removed within one day (60.1%) or a week of the complaint (28,5%), as also reported in other studies2., 7., 13.. Only 4.4% of the patients required general anesthesia or sedation to have the FB removed, with most procedures being carried out in an operating room. The observed failure rate and consequent need for general anesthesia to remove the foreign bodies was lower than the rates reported in the literature, according to which general anesthesia is needed in 30%2., 9., 13. of the cases. Only thirty-seven cases had complications (4.5%). Other studies have reported complication rates as high as 22.2%1., 3., 7., 12., 14..

The differences in relation to the literature seen both in the need for general anesthesia and complication rates may be explained by the fact that our cases of FB were seen only by ENT physicians. As experts on the matter, they are more used to properly managing cases of FB, which by its turn reduces the chances of complication4., 7.. Nonetheless, one should not ignore the limitations of this retrospective study, conducted based on the digital charts of the patients seen in our service.

Patients with myiasis had to return several times for reassessment and follow-up. The treatment of myiasis consists of mechanically removing all larvae and administering antibiotics for often present secondary infection. Treatment includes oral ivermectin and 2% topical iodoform when needed15., 16.. Myiasis has been correlated with necrotic cavity lesions such as middle ear cholesteatoma, tumor, and nasal ulcerogranulomatous diseases (i.e., leishmaniasis, hanseniasis), and oral tumors, in addition to socioeconomic factors and psychiatric disease15., 16..

## CONCLUSION

Foreign bodies are a common occurrence in the practice of otorhinolaryngology. Most foreign bodies are found in patient ears, and children are the most affected age group. Despite the methodological limitations of this study, low complication rates and little need for general anesthesia were verified in our service, as foreign bodies were removed exclusively by ENT physicians. The proper management of foreign bodies requires the aid of specialized physicians.

## References

[1] Figueiredo RR, Azevedo AA, Kós AO, Tomita S. (2008). Complications of ENT foreign bodies: a retrospective study. Braz J Otorhinolaryngol.

[2] Silva BSR, Souza LO, Camera MG, Tamiso AGB, Castanheira LVR. (2009). Foreign bodies in otorhinolaryngology: a study of 128 cases. Int Arch Otorhinolaryngol.

[3] Bressler K, Shelton C. (1993). Ear foreign-body removal: a review of 98 consecutive cases. Laryngoscope.

[4] Mukherjee A, Haldar D, Dutta S, Dutta M, Saha J, Sinha R. (2011). Ear, nose and throat foreign bodies in children: a search for socio-demographic correlates. Int J Pediatr Otorhinolaryngol.

[5] Heim SW, Maughan KL. (2007). Foreign bodies in the ear, nose, and throat. Am Fam Physician.

[6] Thompson SK, Wein RO, Dutcher PO. (2003). External auditory canal foreign body removal: management practices and outcomes. Laryngoscope.

[7] Tiago RS, Salgado DC, Corrêa JP, Pio MR, Lambert EE. (2006). Foreign body in ear, nose and oropharynx: experience from a tertiary hospital. Braz J Otorhinolaryngol.

[8] Marin JR (2006). Trainor JL Foreign body removal from the external auditory canal in a pediatric emergency department. Pediatr Emerg Care.

[9] Ansley JF, Cunningham MJ. (1998). Treatment of aural foreign bodies in children. Pediatrics.

[10] Endican S, Garap JP, Dubey SP. (2006). Ear, nose and throat foreign bodies in Melanesian children: an analysis of 1037 cases. Int J Pediatr Otorhinolaryngol.

[11] Das SK (1984). Aetiological evaluation of foreign bodies in the ear and nose. J Laryngol Otol.

[12] Balbani AP, Sanchez TG, Butugan O, Kii MA, Angélico FV, Ikino CM (1998). Ear and nose foreign body removal in children. Int J Pediatr Otorhinolaryngol.

[13] Ikino CMY, DAntonio WEPA, Balbani APS, Sanchez TG, Butugan O. (1998). Análise dos atendimentos para retirada de corpos estranhos de ouvido e nariz em crianças. Rev Bras Otorrinolaringol.

[14] Schulze SL, Kerschner J, Beste D. (2002). Pediatric external auditory canal foreign bodies: a review of 698 cases. Otolaryngol Head Neck Surg.

[15] Antunes AA, Santos Tde S, Avelar RL, Martins Neto EC, Macedo Neres B, Laureano Filho JR. (2011). Oral and maxillofacial myiasis: a case series and literature review. Oral Surg Oral Med Oral Pathol Oral Radiol Endod.

[16] Arora S, Sharma JK, Pippal SK, Sethi Y, Yadav A. (2009). Clinical etiology of myiasis in ENT: a reterograde period—interval study. Braz J Otorhinolaryngol.

